# Long Term Results and Prognostic Biomarkers for Anti-PD1 Immunotherapy Used after BRAFi/MEKi Combination in Advanced Cutaneous Melanoma Patients

**DOI:** 10.3390/cancers14092123

**Published:** 2022-04-24

**Authors:** Paweł Rogala, Anna M. Czarnecka, Bożena Cybulska-Stopa, Krzysztof Ostaszewski, Karolina Piejko, Marcin Ziętek, Robert Dziura, Ewa Rutkowska, Łukasz Galus, Natasza Kempa-Kamińska, Joanna Seredyńska, Wiesław Bal, Katarzyna Kozak, Anna Surus-Hyla, Tomasz Kubiatowski, Grażyna Kamińska-Winciorek, Rafał Suwiński, Jacek Mackiewicz, Piotr Rutkowski

**Affiliations:** 1 Department of Soft Tissue/Bone Sarcoma and Melanoma, Maria Sklodowska-Curie National Research Institute of Oncology, 02-781 Warsaw, Poland; pawel.rogala@pib-nio.pl (P.R.); krzysztof.ostaszewski@pib-nio.pl (K.O.); katarzyna.kozak@pib-nio.pl (K.K.); piotr.rutkowski@pib-nio.pl (P.R.); 2 Department of Experimental Pharmacology, Mossakowski Medical Research Centre, Polish Academy of Sciences, 02-106 Warsaw, Poland; 3 Department of Clinical Oncology, Maria Sklodowska-Curie National Research Institute of Oncology, Cracow Branch, 31-115 Kraków, Poland; bozena.cybulska-stopa@onkologia.krakow.pl (B.C.-S.); karolina.wojdyla@hotmail.com (K.P.); joanna.seredynska@onkologia.krakow.pl (J.S.); 4 Department of Surgical Oncology, Wroclaw Comprehensive Cancer Center, 53-413 Wroclaw, Poland; zietek.marcin@dco.com.pl; 5 Department of Oncology, Wroclaw Medical University, 50-376 Wroclaw, Poland; 6 Department of Clinical Oncology, Holy Cross Cancer Center, 25-734 Kielce, Poland; robert.dziura@onkol.kielce.pl (R.D.); ewarutkowskasco@gmail.com (E.R.); 7 Department of Medical and Experimental Oncology, University of Medical Sciences, 61-701 Poznan, Poland; galus.lukasz@spsk2.pl (Ł.G.); jmackiewicz@ump.edu.pl (J.M.); 8 Department of Clinical Oncology, Wroclaw Comprehensive Cancer Center, 53-413 Wroclaw, Poland; kempa.natasza@dco.com.pl; 9 Department of Chemotherapy, Maria Sklodowska-Curie National Research Institute of Oncology, Gliwice Branch, 44-102 Gliwice, Poland; wieslaw.bal@io.gliwice.pl; 10 Clinical Department of Oncology and Immuno-Oncology, Warmian-Masurian Cancer Center of The Ministry of The Interior and Administration’s Hospital, 10-228 Olsztyn, Poland; a.surus@wp.pl (A.S.-H.); ord.chemioterapia@poliklinika.net (T.K.); 11 The Skin Cancer and Melanoma Team, Department of Bone Marrow Transplantation and Hematology-Oncology, Maria Sklodowska-Curie National Research Institute of Oncology, Gliwice Branch, 44-102 Gliwice, Poland; grazyna.kaminska-winciorek@io.gliwice.pl; 12 II Clinic of Radiotherapy and Chemotherapy, Maria Sklodowska-Curie National Research Institute of Oncology, Gliwice Branch, 44-102 Gliwice, Poland; rafal.suwinski@io.gliwice.pl; 13 Department of Diagnostics and Cancer Immunology, Greater Poland Cancer Centre, 61-866 Poznan, Poland

**Keywords:** melanoma, immunotherapy, nivolumab, pembrolizumab, BRAF

## Abstract

**Simple Summary:**

Second line anti-PD1 immunotherapy is effective in selected *BRAF*-mutated melanoma patients after BRAFi/MEKi immunotherapy failure. Nivolumab and pembrolizumab are of similar efficacy as a second line therapy. Worse performance status as well as high LDH levels are negative clinical biomarkers that correlated with shorter OS. The presence of brain and liver metastases correlates with shorter PFS and OS of these patients.

**Abstract:**

(1) Background: BRAFi/MEKi are usually offered as a first line treatment for patients requiring rapid response; with elevated lactate dehydrogenase (LDH) activity, large tumor burden, and with brain metastases. The efficacy of second line therapies after BRAFi/MEKI failure is now well defined. (2) Methods: Patients treated with first line target BRAFi/MEKi therapy (vemurafenib plus cobimetinib, dabrafenib plus trametinib or encorafenib plus binimetinib); and for the second line treatment immunotherapy with programmed cell death 1 (PD-1) checkpoint inhibitors (nivolumab or pembrolizumab) with at least one cycle of second line were analyzed for survival and prognostic biomarkers. (3) Results: There were no statistically significant differences in ORR between the treatment groups with nivolumab and pembrolizumab, as well as median progression free-survival (PSF) and overall survival (OS) since the initiation of second line therapy; on nivolumab OS was 6.6 months, and on pembrolizumab 5.0 months. The greatest clinical benefit with second line immunotherapy was observed in patients with LDH ≤ ULN and <3 organ sites with metastasis at baseline. Longer OS was also noted in patients with time to PD  >6 months in first line (slow progression). (4) Conclusions: Second line anti-PD1 immunotherapy is effective in BRAF-mutated melanoma patients after BRAFi/MEKi therapy failure.

## 1. Introduction

Currently, patients with *BRAF* (v-Raf murine sarcoma viral oncogene homolog B)-mutated melanoma patients are offered immunotherapy (pembrolizumab, nivolumab, or nivolumab and ipilimumab combination) or BRAF and MEK (Mitogen-Activated Protein Kinase Kinase 1, *MAP2K1*) inhibitors combination (BRAFi/MEKi) as a first line therapy [1,2,3,4]. Optimal first line therapy has not been clearly defined yet, although primary results of SECOMBIT and DREAMSeq trials suggest that in those individuals who qualify for combination immunotherapy, with nivolumab and ipilimumab, this could be a preferred choice [5,6,7]. At the same time, not all patients are good candidates for combination immunotherapy, and the toxicity of the doublet is relatively high with grade 5 also reported, as confirmed in phase III CheckMate069 and the expanded access program CheckMate 218 [8,9]. The choice of BRAFi/MEKi (vemurafenib and cobimetinib, dabrafenib and trametinib, or encorafenib and binimetinib) is favored by objective response rates (ORR) of more than 65%, oral administration, and low number of outpatient visits, which is of special interest in COVID pandemic [10,11,12,13]. Therefore BRAFi/MEKi anti-PD-1 immunotherapy sequential treatment is actually offered to large group of patients in routine clinical practice [14].

*BRAF*-mutated melanomas were shown as immunologically cold tumors with a downregulated expression of major histocompatibility complex (MHC) class I molecules, low effector T-cell infiltration, and high regulatory T-cells (Tregs), high myeloid-derived suppressor cells (MDSCs) infiltrates, as well as the accumulation of immunosuppressive interleukins (IL-6, IL-10) in the tumor niche, as well as impaired maturation of dendritic cells (DC) and their capacity to secrete proinflammatory cytokines (IL-12 and TNFα) [15,16,17]. On the contrary, multiple studies have shown that mutant BRAF protein epitopes may be recognized by host immunity and induce anti-melanoma immune responses [17,18,19]. Moreover, longitudinal studies have shown that BRAFi/MEKi treatment influence immune status of melanoma tumors. First molecular analyses have shown that initially treatment with either BRAF monotherapy or BRAF and MEK inhibitors combination (BRAFi/MEKi) initially promote the high expression of melanoma antigen recognized by T-cells 1 (MART-1, MLANA), tyrosinase-related protein 1 and 2 (TYRP-1 and TYRP-2), and melanocyte protein PMEL (GP100), namely melanoma antigens, and promote CD8+ T cell infiltration into the tumor niche. At the same time, the downregulation of interleukin-6 (IL-6)- and IL-8, namely immunosuppressive cytokines, and an increase of perforin and granzyme B expression, namely markers of T cell cytotoxicity in the tumors, was found. On the contrary, the expression of exhaustion markers, namely T cell immunoglobulin and mucin domain-containing protein 3 (TIM-3), and programmed cell death protein 1 (PD1), as well as of the immunosuppressive ligand programmed death-ligand 1 (PDL1), increase on BRAFi treatment. Further, at time of disease progression (PD), the downregulation of melanoma antigen expression and CD8 T cell infiltrate decrease was reported on BRAFi therapy [20]. During BRAFi treatment, in the PLX4720 model, melanoma gradually tumors developed immunosuppressive phenotype. The accumulation of regulatory T cells (Tregs) and CD11b(+)/Gr-1(+) myeloid cells as well as the loss of Th1 effector functions of CD4(+) tumor-infiltrating lymphocytes (TILs), including CD40L and interferon gamma (IFNɣ) expression, was observed [15]. Subsequently, BRAF inhibition results in paracrine suppressive activity of melanoma cells on dendritic cells by inhibiting their excretion of pro-inflammatory cytokines, namely IL-12 and tumor necrosis factor alpha (TNFα) [16].

At this point in time, limited data have been published concerning sequential treatment efficacy in patients with BRAF-mutant melanoma. Selected reports suggest lower activity of immunotherapy after PD on BRAFi/MEKi [21,22]. In the DREAMseq trial, response rates were similar for dabrafenib plus trametinib whether used in first or second line, while nivolumab with ipilimumab has shown lower ORR in second line, after PD on dabrafenib–trametinib first-line therapy [23]. Reported ipilimumab monotherapy ORR are lower after BRAFi failure than in the first line [24], while the efficacy of nivolumab after BRAFi or BRAFi/MEKi therapy was reported not to be changed [25]. In general, as it is often suggested that first line treatment with targeted therapy may select aggressive and low-immunogenic melanoma cells, second line immunotherapy may not be as effective [26]. Therefore, BRAFi/MEKi are usually offered as first line treatment for patients requiring rapid response; with elevated LDH activity, large tumor burden, and with brain metastases [27].

Real-world data analysis may provide information on the actual efficacy of nivolumab and pembrolizumab used as a second line treatment, after BRAFi/MEKi treatment. Analysis of regular clinical practice provides insight on the PFS, OS, and ORR in patients who did not qualify for clinical trials. In fact, a low number of analyses describe the long-term survival of *BRAF* mutated melanoma patients and the efficacy of second- or third-line therapy [14,28]. Moreover, second line treatment trials are currently rarely conducted in the melanoma field. Therefore, the analysis of patients who completed sequential treatment may help identify individuals who benefit from currently available melanoma BRAFi/MEKI–anti-PD1 sequential therapy. To date, no such analysis is available. The primary objectives of this study are to analyze the efficacy of anti-PD1 immunotherapy as a second line therapy in patients with unresectable and advanced melanoma. The secondary aim of the study is to characterized patients with advanced melanoma who may benefit from treatment with a targeted therapy -> immunotherapy sequence. Results of our analysis are expected to support the selection of sequential therapies in everyday practice.

## 2. Materials and Methods

### 2.1. Patients

For this observational study, we analyzed health record data of adult patients who started first-line therapy for advanced/metastatic *BRAF*-mutated melanoma between 1 December 2015 and 31 December 2020. The observation data cut off was 31 January 2022. We have included all consecutive sequentially treated patients from major oncology centers in Poland who have been treated with first line target BRAFi/MEKi therapy (vemurafenib plus cobimetinib, dabrafenib plus trametinib, or encorafenib plus binimetinib); and for the second line treatment immunotherapy with anti-PD-1 checkpoint inhibitors (nivolumab or pembrolizumab) with at least one cycle of second line therapy. Patients, if presented with brain metastases, were asymptomatic at treatment initiation and did not require steroid of >10 mg prednisone treatment when immunotherapy was started. All eligible patients had their diagnosis confirmed by pathologists experienced in skin cancer pathology and confirmed BRAF mutation. Patients were treated as described before [29,30] until RECIST 1.1 PD or death whichever comes first. Patients treated with neoadjuvant [31], adjuvant therapies, and within clinical trials were excluded from the study. Date of death was confirmed in the Polish National Cancer Registry via the personal identification number of all patients.

### 2.2. Data Analysis

Descriptive statistics were used to characterize baseline demographic features, including disease stage, metastases loci, as well as treatment duration and best response. Progression free survival (PFS) and OS were calculated with the Kaplan–Meier method, and a log-rank test was used for assessing differences between survival curves. The Cox proportional hazard model was used for multivariable analysis. All variables with a *p*-value < 0.1 in univariate analysis were included in the multivariable model, and 95% confidence intervals (CI) were reported. The differences were considered statistically significant if the *p*-values were <0.05 [32]. Patients without signs of PD were censored at the last follow-up visit. OS was calculated from the date of treatment start to death or last follow-up. Analysis was performed with Statistica version 13.3.

## 3. Results

### 3.1. Patients Treated

The enrolled patients included 106 (51%) females and 101 (49%) males (Table 1) with median age of 57 years (24–89 years old.). A majority (94%) of patients started treatment with stage IV disease, and among all patients almost 23% presented with asymptomatic brain metastases at treatment initiation. More than 61% of patients had elevated LDH at first line treatment start. Median follow-up time from initiation of first line therapy was over 16.8 months and after second line immunotherapy 5.1 months.

### 3.2. Sequential Treatment

**Figure 1:**
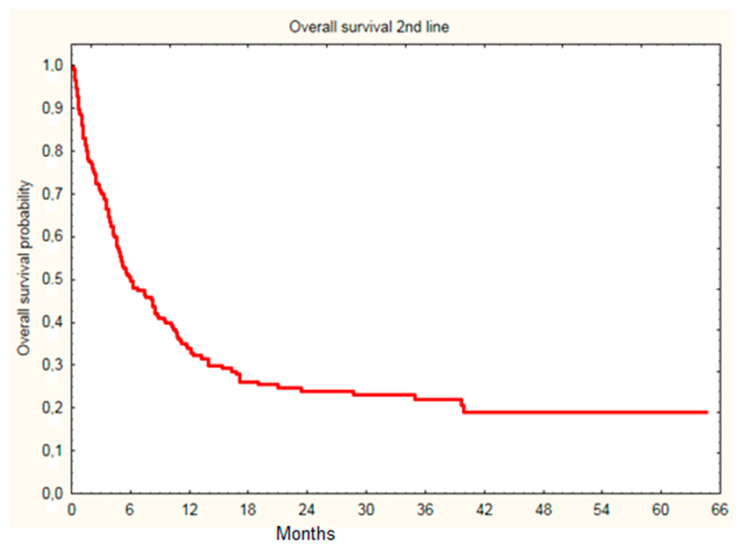
Overall survival of melanoma patients since initiation of second line immunotherapy (nivolumab or pembrolizumab) treatment (18.7 month median).

In the whole group, the most common first-line treatment used was dabrafenib and trametinib combination (141 cases, 68% of all treated patients), while encorafenib and binimetinib was used in only two patients. Among patients treated with immunotherapy in second line treatment, 33 patients continued treatment at the time of analysis. At data cut-off, 11 patients were treated with third line therapy, 11 patients were referred for BSC due to PD, and 148 patients died. Median OS since first line treatment initiation was 18.7 month, while 6.0 months since the initiation of immunotherapy (Figure 1).

**Figure 2:**
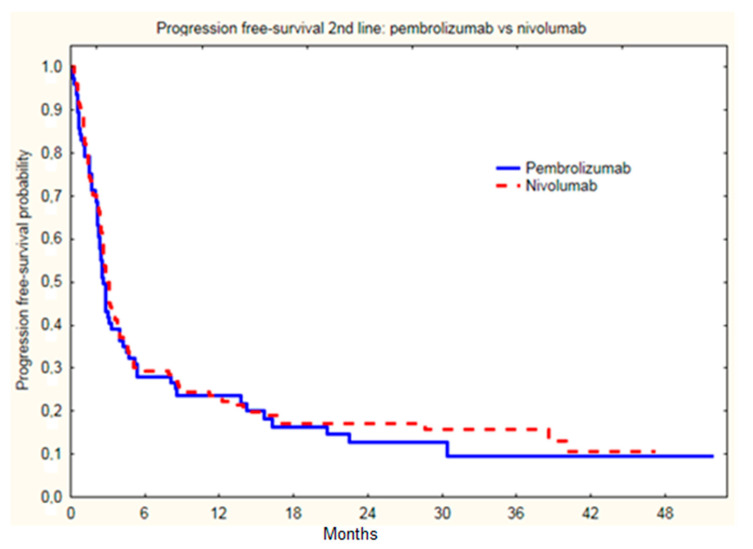
Progression free survival of melanoma patients since initiation of second line nivolumab or pembrolizumab treatment (PSF on nivolumab was 3.0 months, and on pembrolizumab was 2.7 months; *p =* 0.54).

**Figure 3:**
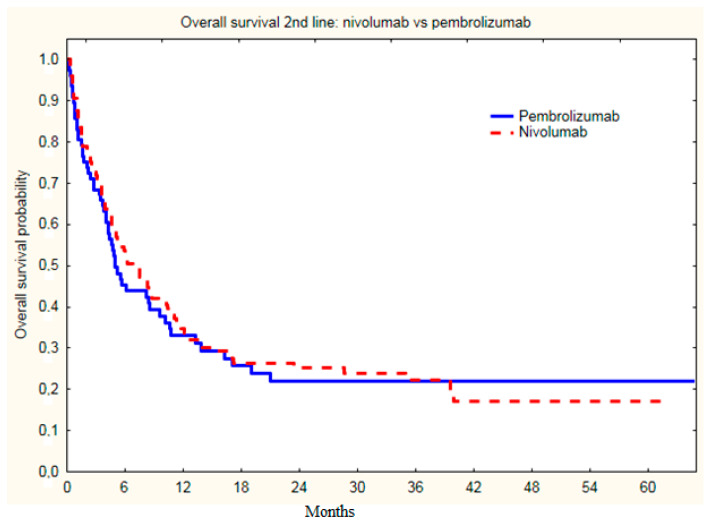
Overall survival of melanoma patients since initiation of second line nivolumab or pembrolizumab treatment. (OS on nivolumab was 6.6 months, and on pembrolizumab was 5.0 months; *p =* 0.99).

There were no statistically significant differences in ORR between the treatment groups with nivolumab and pembrolizumab (*p =* 0.90) (Table 2), as well as median PSF (*p =* 0.54), as PSF on nivolumab was 3.0 months, and on pembrolizumab 2.7 months (Figure 2); and OS on nivolumab was 6.6 months, and on pembrolizumab 5.0 months (Figure 3).

**Figure 4:**
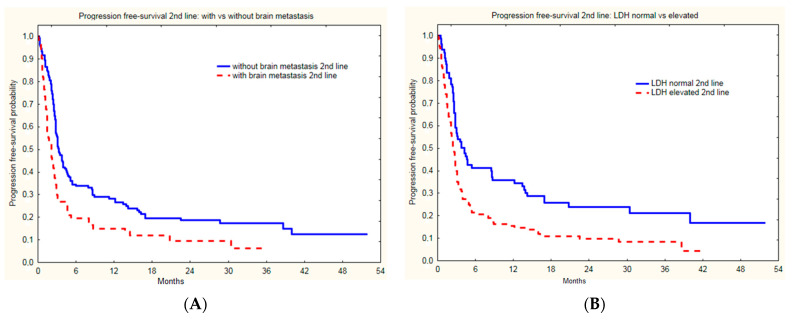
Progression free survival of melanoma patients with and without brain metastases (**A**) and normal and elevated LDH level (**B**) since initiation of second line immunotherapy.

Groups treated with nivolumab and pembrolizumab did not differ in baseline subgroup characteristics based on LDH level (*p =* 0.99), gender (*p =* 0.19), age (*p =* 0.31), or presence of brain metastases (*p =* 0.59). The presence of liver metastases, lower performance status (ECOG1), and high LDH activity correlated with PSF on second line immunotherapy (*p =* 0.0208, *p =* 0.0179 and *p =* 0.0069, respectively) in the whole group (Table 3, Figure 4).

**Figure 5:**
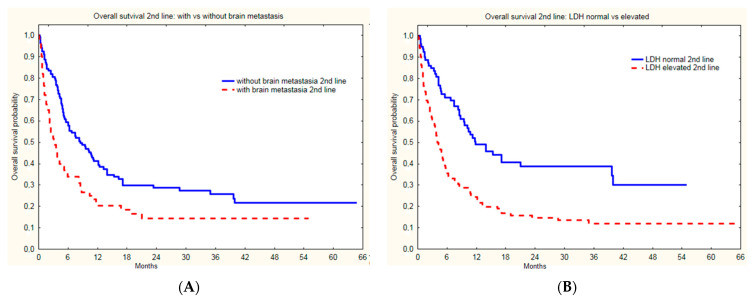
Overall survival of melanoma patients with and without brain metastases (**A**) and normal and elevated LDH level (**B**) since initiation of second line immunotherapy.

The presence of brain metastases (Figure 5A) and LDH level (Figure 5B) correlated with median OS (*p* < 0.0001 and *p* < 0.0001, respectively). Moreover, the presence of liver metastases at the initiation of second line therapy, i.e., immunotherapy, correlated with OS (Table 4).

**Figure 6:**
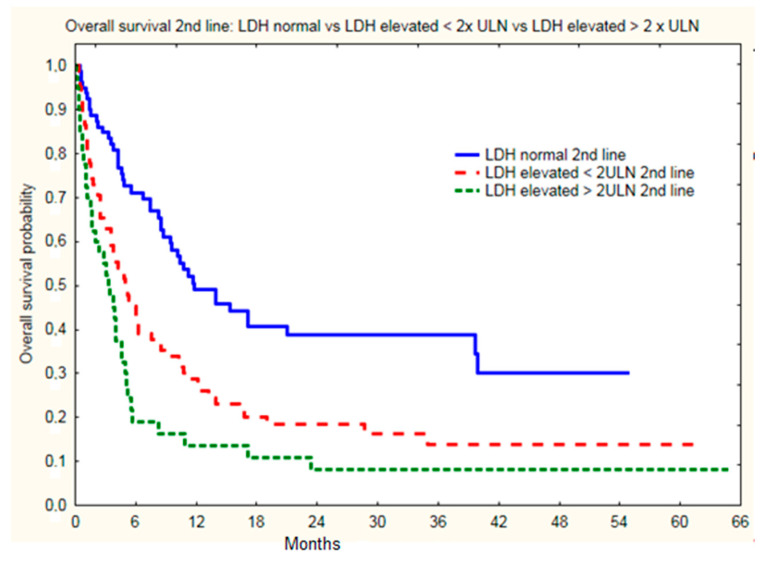
Overall survival of melanoma patients with normal and elevated LDH level since initiation of second line immunotherapy.

For anti-PD1 treatment, statistically significant differences were found in the OS of subgroups of patients with different LDH levels. The highest survival was observed in patients with normal LDH levels, where 24-month OS was 39% vs. 19% for patients with elevated LDH (<2ULN) vs. 8% for patients with LDH > 2ULN (*p <* 0.0001) (Figure 6). Other factors that correlated with OS achieved after initiation of second line treatment were presence with brain metastases both at treatment initiation as well as at second line therapy initiation (Table 4). None of the patients without brain and without liver metastases had normal LDH (Table 5). High LDH level also correlated with shorter survival in patients with liver (*p* = 0.004) metastases and in patients without brain metastases (*p* = 0.007). The greatest clinical benefit with second line immunotherapy and longest OS was observed in patients with LDH ≤ ULN and without liver or brain metastases at baseline (>2 years). Longer OS was also noted in patients with time to PD >6 months in first line (slow progression).

## 4. Discussion

In this analysis, the survival of unresectable and metastatic melanoma patients who received sequential therapy was investigated. Our real-world analysis utilizing nation-wide data from multiple reference melanoma reference centers confirms that treatment with second-line anti-PD-1 therapy prolongs overall survival in selected patients with advanced/metastatic *BRAF*-mutated melanoma, including patients with initially poor performance status. Patients with high tumor burden are likely to benefit from initial response to BRAFi/MEKi therapy and may continue treatment with anti-PD-1 as second line treatment. Our data is supported by other reports [33,34,35]. In the pooled analysis of phase 3 COMBI-d (NCT01584648) and COMBI-v (NCT01597908) trials that included 563 patients who received dabrafenib plus trametinib, 299 patients received subsequent anticancer therapies: 151 (51%) received an anti–CTLA-4 therapy and 102 (34%) received an anti–PD-1 therapy [34]. In a long-term KEYNOTE-006 trial follow up, *BRAF*-mutated melanoma patients who were not treated with a prior BRAFi lad longer PSF on pembrolizumab treatment (7.0 months) than those who were treated initially witb RAFi (2.8 months). Moreover, patients with *BRAF* V600E/K–mutant melanoma who received a previous BRAFi with or without MEKi had lower objective response rates (28.4% vs. 44.2%), four-year PFS (15.2% vs. 27.8%), and OS (26.9% vs. 49.3%), compared with those who had not received earlier targeted therapy [35]. In a phase IIa study (NCT02083354) of patients with unresectable or metastatic BRAF V600-mutant acral/cutaneous melanoma treated with dabrafenib (150 mg twice daily) with trametinib (2 mg once daily), treatment sequencing results were collected. Patients (*n* = 10/53) who were treated with a PD-1 inhibitor after PD on dabrafenib plus trametinib and achieved a median post progression survival (PPS) of 17.6 months (95%CI 16.9–28.3), which is similar to the OS reported by us [33].

Treatment beyond frontline therapy for patients with BRAF-mutated advanced/metastatic melanoma is currently under investigation in prospective trials. A first study that may provide more data is ImmunoCobiVem (NCT02902029), a German phase II trial comparing cobimetinib and vemurafenib followed by atezolizumab at PD versus atezolizumab in first line followed by targeted therapy [36]. In the phase III DREAMseq trial, also known as the ECOG-ACRIN EA6134 trial, treatment with nivolumab with ipilimumab resulted in an absolute 20% improvement in two-year OS over first line BRAFi/MEKi (dabrafenib plus trametinib). At the two-year analysis, the OS rate was 72% for patients treated with nivolumab with ipilimumab in first line and targeted therapy in second line. At the same time, the OS rate was only 52% for patients treated with dabrafenib plus trametinib as fist line therapy and with immunotherapy in second line. First line immunotherapy also led to durable responses. ORR for nivolumab with ipilimumab was 46% and for dabrafenib plus trametinib was 43%. After PD on first line therapy, response rates to second-line therapy were 48% for dabrafenib plus trametinib and 30% for nivolumab with ipilimumab. Response rates were similar for dabrafenib plus trametinib whether used first of second line. On the contrary, nivolumab with ipilimumab was less effective when used in second line. At a median follow-up of 27.7 months, 100 died, 38 patients who had first line of nivolumab with ipilimumab first and 62 in those treated with BRAFi/MEKi, which translates into a 20% difference in survival [23]. In the phase II Sequential Combo Immuno and Target Therapy (SECOMBIT, NCT02631447), treatment with nivolumab with ipilimumab followed by encorafenib plus binimetinib at PD (Arm B) and adopting the ‘sandwich’ strategy starting with encorafenib/binimetinib for eight weeks, then ipilimumab/nivolumab for eight weeks (arm C), improved survival rates over encorafenib plus binimetinib followed by nivolumab with ipilimumab were observed (Arm A). In this trial median OS was not yet reached in any of the arms, but the survival rate at two years was 73% for immunotherapy combination used first (arm B), 65% for the BRAFi/MEKi combination first (arm A), and 69% for the sandwich approach (arm C). At three years, it was 62%, 54%, and 60%, respectively. Moreover, the response rate to immunotherapy combination was 45% in the first line, but 25% in the second line, after PD on BRAFiMEKi [37,38]. Although data on immunotherapy combination use in first line treatment is interesting, we need to remember that not all patients will qualify for immunotherapy combination treatment and selected patients require fast response induction [39]. In the absence of known prognostic or predictive molecular biomarkers to determine the selection of BRAFi/MEKi as first line therapy, clinical factors are used to anticipate disease dynamics. We confirm that after PD, BRAFi/MEKi patients who are in good performance status still benefit from second line therapy. Patients who benefit with objective response on BRAFi/MEKi are expected to obtain additional OS benefit when offered second line therapy.

Real-word evidence, although of lower evidence level than randomized trials, helps to define the effectiveness of treatments in routine clinical practice, including subgroups of patients who are usually excluded or under-represented in the trials [40]. Limitations of real-world analysis could include patient selection bias due to the referral of patients in better performance status to immunotherapy or clinical trials. Moreover, in treatment outside of clinical trial, detailed patient data may be inconsistently collected during long-term observation or between different hospitals. In single cases, data on variables could be missed as a result of data collection bias. Nevertheless, due to the strict regulation of therapy reimbursement in Poland, data regarding melanoma patients (the description of regulations of national drug program is available here: https://www.gov.pl/web/zdrowie/choroby-onkologiczne—B59, accessed on 1 April 2022) must be recorded consistently in the whole country in a central electronic system. Our analysis may provide clinically significant information on second line immunotherapy in real-world practice in the melanoma field as well as prognostic biomarkers in these patients. Experienced researchers, data managers, and melanoma experts with interpretation skills were included in our research team in order to provide the proper interpretation of results. The treatment efficacy described by us complements the results reported by phase III registration trials and covers long-term observations of second line of therapy [40].

In our study, we have confirmed the significance of liver metastases as a negative biomarker for immunotherapy efficacy outside of clinical trials [41], which is expected due to their role in CD8+ T-cell elimination [42]. Moreover, our data support the role of LDH as negative prognostic biomarker in melanoma immunotherapy, namely in that a higher pre-treatment serum LDH level is predictive of shorter PFS [43]. High LDH levels are known to be inversely correlated with response to checkpoint inhibitors because LDH levels are associated with high tumor burden, enhanced glycolytic activity, and hypoxia-induced necrosis in the tumor niche. At the biochemical level, melanoma tumor-derived lactate increases the number of infiltrating MDSC in the tumors, as well as polarizes tumor-associated macrophages into immune suppressive M2 macrophages [44]. Most recent multi-omics analysis of metastatic melanoma patients identified no significant intra-tumoral molecular, immunological, or metabolic associations with serum LDH, including glycolysis, metabolism drivers, glucose metabolism, hypoxia, mTOR pathway, choline metabolism, checkpoint inhibitors expression, or genes of the adaptive and innate immune response. These authors have suggested that serum LDH serves as a surrogate of tumor burden, but not of tumor phenotype. Patients with elevated serum LDH levels had a significantly higher number of metastases in comparison to patients with normal serum LDH levels [45]. At the same time, intra-tumoral lactate dehydrogenase C isoform (LDHC) activation in melanoma cells provides a metabolic rescue pathway for these cells via the preference for lactate metabolism for ATP generation [46,47,48]. Clinical biomarkers seem to be surrogates of low immunity and potential immune-desert tumors, as also hypothesized by other authors [49]. Moreover, in the case of melanoma, more complex prognostic indexes could be developed in the future. At this point in time, the derived neutrophils/(leukocytes minus neutrophils) ratio, so called dNLR, along with LDH level, constitute an immune prognostic index that correlates with immunotherapy treatment outcome [47]. It may be expected that other clinical parameters, e.g., renal function or albumin levels, could be prognostic for immunotherapy, but not other types of treatment [47,48]. Additional translational analyses should be designed to directly measure immune signatures that could potentially correlate with anti-PD1 response after BRAFi/MEKi failure, as initial studies have not yet covered sequential treatment [50,51]. In terms of clinical practice, it may be expected that switching treatment to immunotherapy upon response to BRAFi/MEKi with LDH normalization could be an effective strategy to prolong OS. This is expected to be an effective approach in advanced melanoma patients with initial highly elevated serum LDH [52]. Others suggest that such patients may benefit from triple combination therapy [53,54].

## 5. Conclusions

Second line anti-PD1 immunotherapy is effective in *BRAF*-mutated melanoma patients after BRAFi/MEKi therapy failure. The presence of brain and liver metastases correlated with shorter PFS on second line treatment as well as the total OS of these patients. Poor performance status as well as high LDH levels are also clinical negative biomarkers that correlated with shorter survival. Patients with high tumor burden, including brain metastases, should be offered second line therapy, as about 20% may achieve an objective response, while another 20% may obtain some clinical benefit with disease stabilization. Further prospective studies of sequential treatment should include molecular and translational analysis to define additional predictive and prognostic biomarkers.

## Figures and Tables

**Table 1:** Baseline patients characteristics.

Factor		Patients *n* = 207	Percentage
Sex	F	106	51%
	M	101	49%
Disease stage 1L TNM stage (AJCC 8th Edition)	III Localy advanced	13	6%
	M1a	30	14.5%
	M1b	30	14.5%
	M1c	87	42%
	M1d	47	23%
LDH 1L	Normal	78	39%
	Over ULN	124	61%
	Less than 2× over ULN	80	40%
	More than 2× over ULN	44	21%
	No data	5	-
ECOG 1L	0	77	37%
	1	124	60%
	2	6	3%
Liver metastases 1L	No	137	66%
	Yes	70	34%
Brain metastases 1L	No	160	77%
	Yes	47	23%
First line treatment	Dabrafenib + trametinib	141	68%
	Vemurafenib + cobimetinib	64	31%
	Encorafenib + binimetinib	2	1%
Second line treatment	Nivolumab	130	63%
	Pembrolizumab	77	37%
LDH 2L	Normal	81	39%
	Over ULN	124	61%
	Less than 2× over ULN	84	41%
	More than 2× over ULN	40	20%
	No data	2	-
ECOG 2L	0	33	16%
	1	163	79%
	2	10	5%
	No data	1	-
Liver metastases 2L	No	138	67%
	Yes	69	33%
Brain metastases 2L	No	135	65%
	Yes	72	35%

F, female; M, male; LDH, lactate dehydrogenase; ECOG, Eastern Cooperative Oncology Group (performance status); TNM, tumor, node, metastasis (staging system); AJCC, American Joint Committee on Cancer; 1L, first line treatment; 2L, second line treatment.

**Table 2:** Best response on two lines of treatment.

Treatment Response		Patients	Percentage	Patients	Percentage	*p*-Value
Total		1L		2L		
Best response	PD	21	10%	123	61%	<0.0001
	SD	71	34%	39	19%	
	PR	106	51%	32	16%	
	CR	9	5%	9	4%	
	ORR	196	56%	41	20%	<0.0001
	Not assessed	0	-	4	-	-
Time to PD	>6 m	137	66%	57	28	-
	<6 m	70	34%	144 *	72%	-
2L		Nivolumab		Pembrolizumab		
Best response	PD	76	60%	47	62%	0.99
	SD	25	20%	14	18%	
	PR	20	16%	12	16%	
	CR	6	4%	3	4%	
	ORR	26	20%	15	20%	0.90
	Not assessed	3	-	1	-	-

CR, complete response; PR, partial response; SD, stable disease; PD, progression disease; ORR (CR + PR), overall response rate; PD, progressive disease; 1L, first line (treatment); 2L, second line (treatment). * 6 patient continuing treatment without progression < 6 months.

**Table 3:** Factors that influence median PFS on second line immunotherapy in advanced metastatic melanoma patients.

	Univariate Analysis			Multivariate Analysis		
Factor	HR	CI 95%	*p*-Value	HR	CI 95%	*p*-Value
Age	1.0	0.9–1.0	0.6772	-		
Sex	0.91	0.7–1.2	0.5350	-		
LDH over ULN 2L	0.57	0.4–0.8	0.0005	0.62	0.4–0.9	0.0069
ECOG 0 2L	0.24	0.1–0.5	0.0004	1.51	0.5–4.5	0.9237
ECOG 1 2L	0.42	0.2–0.8	0.4320	2.42	0.8–6.9	0.0179
Brain metastases 2L	0.58	0.4–0.8	0.0008	0.77	0.5–1.1	0.1807
Liver metastases 2L	0.55	0.4–0.8	0.0002	0.66	0.5–0.9	0.0208
ORR 1L	0.74	0.5–1.0	0.054	0.84	0.6–1.2	0.3408
Time to PD 1L > 6 m	1.49	1.1–2.0	0.0115	1.38	1.0–1.9	0.0666

LDH, lactate dehydrogenase; ECOG, Eastern Cooperative Oncology Group (performance status); PD, progression disease; ORR (CR + PR), overall response rate; PD, progressive disease; 1L, first line (treatment); 2L, second line (treatment); m, months, HR, hazard ratio; CI confidence interval.

**Table 4:** Factors that influence OS in metastatic melanoma patients treated with BRAFi/MEKi -> immunotherapy—treatment sequence.

Factor	Univariate Analysis		Multivariate Analysis			
	HR	CI 95%	*p*-Value	HR	CI 95%	*p*-Value
Age	1.00	1.0–1.0	0.9848	-		
Sex	1.14	0.8–1.6	0.4373	-		
Brain metastases 1L	0.57	0.4–0.8	0.0310	0.62	0.4–0.9	0.0165
Liver metastases 1L	0.70	0.5–1.0	0.0406	0.88	0.6–1.3	0.4954
Brain metastases 2L	0.58	0.4–0.8	0.0011	0.44	0.3–0.6	<0.0001
Liver metastases 2L	0.56	0.4–0.8	0.0007	0.69	0.5–1.0	0.0340
LDH over ULN 1L	0.61	0.4–0.9	0.0042	0.54	0.4–0.8	0.0018
LDH over ULN 2L	0.45	0.3–0.6	<0.0001	0.49	0.3–0.7	<0.0001
Objective response in 1L	0.79	0.6–1.1	0.1421	0.87	0.6–1.4	0.4326
Objective response in 2L	0.14	0.1–0.3	<0.0001	0.13	0.1–0.2	<0.0001
Time to PD 1L > 6 m	2.64	1.9–3.7	<0.0001	2.8	1.9–4.1	<0.0001

F, female; M, male; LDH, lactate dehydrogenase; *ECOG,* Eastern Cooperative Oncology Group (performance status); PD, progression disease; ORR (CR + PR), overall response rate; PD, progressive disease; 1L, first line (treatment); 2L, second line (treatment); m, months, HR, hazard ratio; CL, confidence interval.

**Table 5:** LDH influence on OS in metastatic melanoma patients treated with BRAFi/MEKi -> immunotherapy—treatment sequence.

LDH	Metastasis		Patients (*n*)	Median OS (Months)
LDH normal	liver	without metastasis	57	21.7
		with metastasis	19	21.0
	brain	without metastasis	69	25.2
		with metastasis	9	18.8
	liver and brain	without metastasis	48	24.4
		with metastasis	0	-
LDH elevated	liver	without metastasis	68	16.2
		with metastasis	50	12.2
	brain	without metastasis	87	16.3
		with metastasis	37	9.0
	liver and brain	without metastasis	49	19.0
		with metastasis	18	7.6

LDH, lactate dehydrogenase; OS, overall survival.

## Data Availability

All data are available for research cooperation purposes from the PI of the study upon DTA approval.
